# Metabolic determinants in *Listeria monocytogenes* anaerobic listeriolysin O production

**DOI:** 10.1007/s00203-017-1355-4

**Published:** 2017-03-13

**Authors:** Nathan Wallace, Eric Newton, Elizabeth Abrams, Ashley Zani, Yvonne Sun

**Affiliations:** Department of Biology, University of Dayton, 300 College Park, Dayton, OH 45469 USA

**Keywords:** Anaerobic metabolism, Virulence regulation, Tricarboxylic acid cycle

## Abstract

*Listeria monocytogenes* is a human pathogen and a facultative anaerobe. To better understand how anaerobic growth affects *L. monocytogenes* pathogenesis, we first showed that anaerobic growth led to decreased growth and changes in surface morphology. Moreover, compared to aerobically grown bacteria, anaerobically grown *L. monocytogenes* established higher level of invasion but decreased intracellular growth and actin polymerization in cultured cells. The production of listeriolysin O (LLO) was significantly lower in anaerobic cultures—a phenotype observed in wild type and isogenic mutants lacking transcriptional regulators SigB or CodY or harboring a constitutively active PrfA. To explore potential regulatory mechanisms, we established that the addition of central carbon metabolism intermediates, such as acetate, citrate, fumarate, pyruvate, lactate, and succinate, led to an increase in LLO activity in the anaerobic culture supernatant. These results highlight the regulatory role of central carbon metabolism in *L. monocytogenes* pathogenesis under anaerobic conditions.

## Introduction

*Listeria monocytogenes* is a foodborne pathogen and a leading cause of death from foodborne illnesses (Scallan et al. 2011). While immuno-competent individuals may develop mild gastroenteritis after ingestion of large amounts of *L. monocytogenes*, immuno-compromised individuals have a higher risk of developing systemic infections. These infections can cause more severe symptoms and lead to fatal outcomes despite early antibiotic treatments. Therefore, there is a need to better understand *L. monocytogenes* behavior during transmission to develop effective strategies to prevent infections. Upon ingestion, *L. monocytogenes* transits through the gastrointestinal tract and must adapt to host lumenal conditions to establish infections. However, despite the fact that the intestinal lumen is characterized by varying degrees of oxygenation (He et al. 1999), most of our understanding of *L. monocytogenes* pathogenesis is based on research conducted under aerobic conditions. The extent and the mechanism by which anaerobic exposure impacts *L. monocytogenes* pathogenesis are unclear.

As a facultative anaerobe, *L. monocytogenes* can grow under strict anaerobic conditions with altered carbon metabolism. Chemical analyses have shown that in the presence of oxygen, *L. monocytogenes* incompletely oxidizes glucose to acetate, lactate, and acetoin. In the absence of oxygen, *L. monocytogenes* produces lactate as its major fermentation product along with ethanol, formate, and carbon dioxide (Pine et al. 1989; Romick et al. 1996; Romick and Fleming 1998; Jydegaard-Axelsen et al. 2004). Moreover, transcriptional analyses using *L. monocytogenes* strain EGD showed a decreased transcript level for genes encoding pyruvate dehydrogenase and those involved in acetoin synthesis under anaerobic conditions (Müller-Herbst et al. 2014). Genes encoding phosphotransferases systems also exhibited differential transcript levels in response to suboxic conditions (Toledo-Arana et al. 2009). Together, these studies suggest that oxygen levels play a key role in regulating carbon metabolism in *L. monocytogenes*. However, it is not clear whether or how these metabolic adaptations influence *L. monocytogenes* pathogenesis under anaerobic conditions.

*L. monocytogenes* is an intracellular pathogen capable of growing and spreading between the cytosol of mammalian host cells. Its ability to invade non-phagocytic cells contributes to invasion of intestinal epithelium and subsequent systemic infections. Available evidence suggests that anaerobic growth results in an enhanced invasion phenotype (Bo Andersen et al. 2007; Burkholder et al. 2009). However, the subsequent intracellular growth in the aerobic host cytosol is not known. Moreover, the signals mediating the anaerobic effects on *L. monocytogenes* infection have not been established. In this study, to provide a better understanding of *L. monocytogenes* behavior under anaerobic conditions, we investigated how anaerobic growth and the associated signals from anaerobic metabolism affect *L. monocytogenes* pathogenesis.

## Materials and methods

### Bacterial strains and culture conditions

Culture of the wild type and isogenic mutants of *L. monocytogenes* strain 10403s were grown from colonies on a freshly streaked brain–heart infusion (BHI) plate (<1 week) at 37 °C. Mutants used in this study include those with clean deletion in *sigB* (∆*sigB*) and *codY* (∆*codY*) and one with a constitutively active PrfA (PrfA*) (Bruno and Freitag 2010). All cultures were grown in filter-sterilized BHI media (Lot 4176589) to ensure consistency. Buffered BHI was prepared using 100 mM MOPS buffered at pH 7.0. Aerobic cultures were grown with agitation at 250 RPM to ensure adequate oxygen diffusion. Anaerobic cultures were grown in a temperature-controlled incubator inside an anaerobic chamber (Coy Laboratory, Type A) with a nitrogenous atmosphere containing 2.5% hydrogen. Optical density (OD) was measured in an optically clear 96-well plate at 600 nm with a volume of 200 µL per well using a 96-well plate reader (Biotek Synergy4). Supplements included sodium acetate (Fisher Scientific BP334-500), sodium fumarate (Acros Organics AC21553-1000), sodium succinate (Acros Organics AC20874-5000), sodium citrate (Fisher Scientific S279-500), acetoin (Acros Organics AC 41195-100), sodium pyruvate (Alfa Aesar A11148), and lithium lactate (Acros Organics 413331000). All supplements were prepared as 1 M stock solutions in deionized water, filter-sterilized, and added directly to the media to the desired concentration before inoculation.

### Measurement of lactate, acetoin, and ethanol concentrations

Supernatant lactate concentration was measured using a commercially available enzymatic kit following the manufacturer’s suggested protocol (Fisher 50-489-257). The Voges–Proskauer test (Nicholson 2008) was adapted to quantify acetoin production in the supernatant of overnight *L. monocytogenes* cultures. A supernatant or standard sample (100 µL) was placed into a sterile micro-centrifuge tube followed by additions of 70 µL of 0.5% creatine monohydrate (Sigma C3630-100G), 100 µL of 1-Napthol (Sigma N1000-10G), and 100 µL of 40% KOH (Chempure 831-704) in 95% EtOH. Samples were centrifuged between each addition, and incubated at room temperature for 15 min after the final addition. After incubation, 200 µL of each sample was placed into a flat bottom 96-well plate and the absorbance was read at 560 nm. A standard curve was constructed to calculate the concentration of acetoin in culture supernatant samples. Ethanol percentage was measured using a commercially available enzymatic kit following manufacturer’s suggested protocol (Fisher 50-489-254).

### Transmission electron microscopy

Overnight aerobic and anaerobic cultures of *L. monocytogenes* were visualized using transmission electron microscopy (TEM). Bacterial cultures (3 mL) were spun down to collect pellets, which were first fixed using 2 mL of a 2% paraformaldehyde (Alfa Aesar 30525-89-4) and 2% glutaraldehyde (Alfa Aesar 111-30-8) in phosphate buffer solution for 24 h at 4 °C. Following fixation, cells were washed three times for 10 min in phosphate buffer. Washed cells were then post fixed using a 2% solution of OsO_4_ in phosphate buffer for 24 h at 4 °C. Following post fixation, cells were stained with 2% lead citrate in a phosphate buffer solution at 4 °C for 24 h. After staining, the cells were treated to a series of dehydrations in ethanol (30, 40, 50, 60, 70, 80, 90, 95, and 100%) each for 10 min. The dehydrated cells were then embedded in API-PON 812 epoxy resin monomer (SPI-CHEM 90529-77-4) and dried for 24 h at 70 °C in an oven. The dried samples were sectioned using an ultra-microtome with a diamond blade to 100 nm sections. The sections were then embedded on lacy carbon grids and read using a Hitachi H-7600 Transmission Electron Microscope at 120 kv. Measurements of cell envelope thickness were made using GNU Image Manipulation Program (GIMP).

### Cell culture infection

The murine peritoneal macrophages RAW 264.7 (ATCC TIB-71), Caco-2 colorectal adenocarcinoma cells (ATCC HTB-37), and LS174T mucin-secreting colorectal adenocarcinoma cells (ATCC CL-188) were grown in DMEM (Thermo Scientific SH30285.01) supplemented with 10% (v/v) heat inactivated fetal bovine serum (JRScientific REF 4365-500, Lot N056-6), HEPES (10 mM), and glutamine (2 mM) in a 37 °C incubator with a 5% CO_2_ atmosphere. Prior to infections, cells were seeded in a 24-well tissue culture plate and grown for 14–18 h. Overnight cultures of *L. monocytogenes* were used for infections at an MOI of 10. Bacteria diluted in cell culture medium were added to each well (500 µL) and incubated for 30 min. Following incubation, media were aspirated and cells were washed twice with sterile DPBS. Fresh media (1 mL per well) containing 10 µg/mL gentamicin stock was added to each well. To enumerate intracellular bacteria, cell culture media were aspirated off and sterile 0.1% (v/v) triton X-100 was added to each well (200 µL per well) to lyse host cells. Lysates were diluted and spread on LB plates. Colonies on plates were counted using an automatic colony counter (Synbiosis aCOLyte 3) after 24–48 h of incubation in a 37 °C incubator.

### Immunofluorescence microscopy

RAW264.7 macrophages were plated onto sterile coverslips (18 by 18 mm) inside 6-well plates at 1 million cells per well in the afternoon prior to infections. Overnight, *L. monocytogenes* cultures were washed twice and diluted in cell culture media for infection at an MOI of 10. At 2 hours post infection (hpi), coverslips were fixed in paraformaldehyde (3.7% in PBS) overnight at 4 °C. For immunofluorescence microscopy, each coverslip was washed with TBS-T (25 mM Tris-HCl, 150 mM NaCl, 0.1% Triton X-100) and blocked with TBS-T with 1% bovine serum albumin (BSA). Anti-*Listeria* serum (1:500 in TBS-T with 1% BSA; Thermo Scientific PA1-30487) was added onto each coverslip and incubated at room temperature for 1 h. Each coverslip was washed in 5 ml of TBS-T prior to incubation with secondary antibodies, phalloidin-iFluor 594 (1:400, abcam ab176757) and AlexaFluor 488-goat anti-rabbit antibody (1:400, abcam ab150077), in TBS-T with 1% BSA. One hundred intracellular bacteria per experimental replicate were scored for the presence or absence of actin clouds.

### Hemolytic assays

Hemolytic assays were performed using overnight culture supernatant samples to measure the activity of listeriolysin O (LLO). Each sample was incubated at room temperature with 0.1 M DTT (5 µL) for 15 min. A positive control (0.4% triton X-100) and a negative control (blank BHI media) were included for each experiment. After incubation, samples were serially diluted using hemolysis buffer containing: dibasic sodium phosphate (35 mM) and sodium chloride (125 mM) brought to pH 5.5 with acetic acid. Defibrinated sheep’s blood (Hemostat Laboratories DSB050) was diluted to a hematocrit of 2% and then added to each sample for a final hematocrit of 1%. Samples were incubated at 37 °C for 30 min. After incubation, all samples were spun down at 2000 RPM for 5 min to pellet intact blood cells. Supernatant lysate (120 µL) was transferred to a flat bottom 96-well plate for OD measurement at 541 nm as an indicator for LLO activity. Hemolytic unit was calculated as the inverse of the dilution factor at which half complete lysis occurred and subsequently normalized with original culture OD measured at absorbance at 600 nm. Samples that did not produce lysis at a level more than half of complete lysis were designated as “Below Detection” for their hemolytic units. Supernatant samples from anaerobic cultures typically generate activities at or slightly above “Below Detection” levels.

### SDS-PAGE, silver staining, and immunoblotting

Samples from overnight cultures of *L. monocytogenes* were used for SDS-PAGE and western blotting. Cultures were normalized by optical density (600 nm) using BHI media and centrifuged to separate supernatant and bacterial cell pellets. Supernatant samples were precipitated with 1% trichloroacetic acid at 4 °C for 1 h. Following precipitation, a cold acetone wash was performed. Both the pellet and supernatant samples were resuspended in 12 µL of 2× sample buffer and heated at 95 °C for 5 min. The samples were then separated via SDS-PAGE (8% acrylamide in the separating gel). Following SDS-PAGE, gels were either subjected to silver staining (Thermo Scientific 24612) following manufacturer’s protocol or proteins in gel were transferred to a PVDF membrane for subsequent immunoblotting using anti-LLO rabbit antibody (1:10,000, abcam ab43018) followed by goat anti-rabbit HRP antibody (1:10,000, abcam ab6721). Bands were visualized using chemilluminescent substrate (BIO-RAD 170-5060) and captured with X-ray films (WorldWide Medical Products 41101002).

## Results

### Characterization of anaerobic growth by *Listeria monocytogenes* strain 10403s

**Fig. 1:**
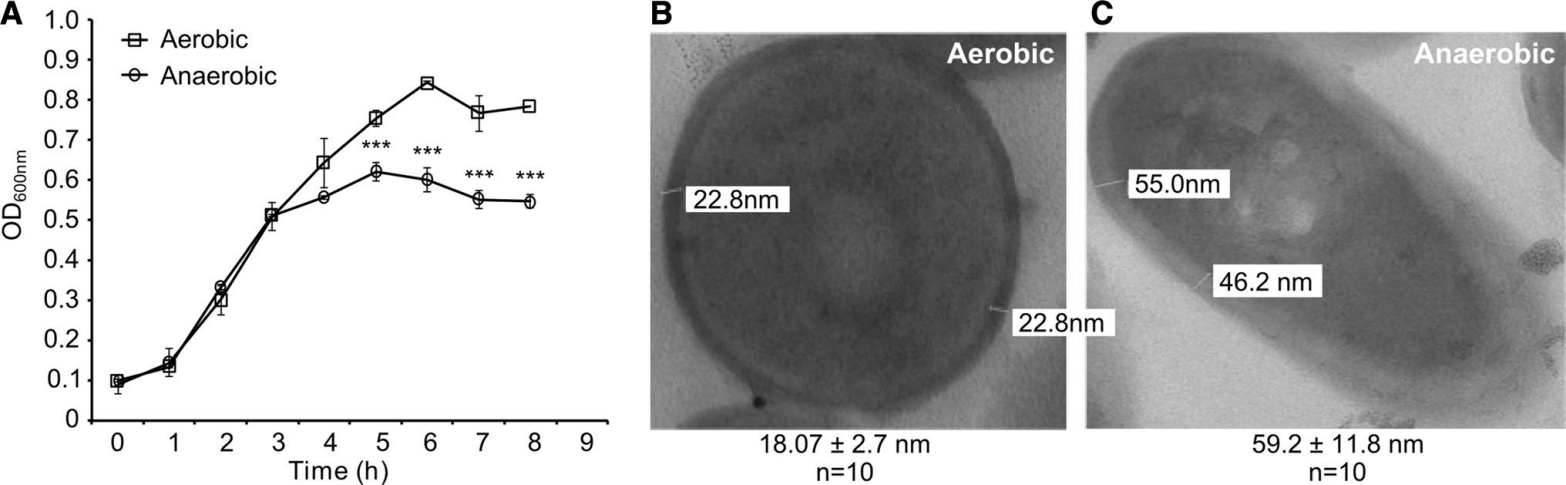
Anaerobically grown *L. monocytogenes* exhibits decreased maximal growth in vitro and morphological differences under TEM. **A** Growth curves of *L. monocytogenes* strain 10403s grown in BHI are plotted on a linear *Y*-axis to show the decreased maximal OD over 8 h of growth. Averages of triplicates are plotted with *error bars* representing the standard deviation and statistics were performed using a two-tailed student’s *t* test with significant differences indicated by asterisks (***p < 0.001). Aerobically (**B**) or anaerobically (**C**) grown *L. monocytogenes* were visualized with TEM. Space between cytoplasm and outer edge of cells (*n* = 10) was measured and shown under their respective images as averages ± standard deviation

Current knowledge of anaerobic metabolism in *L. monocytogenes* is built from research using different laboratory strains (Pine et al. 1989; Romick et al. 1996; Müller-Herbst et al. 2014). Strain 10403s is widely used as a model organism, but its anaerobic metabolism has not been investigated. Therefore, we first monitored in vitro growth of strain 10403s in the presence or absence of oxygen in the standard BHI medium. As expected for a facultative anaerobe, static growth in the absence of oxygen resulted in a lower maximal optical density compared to agitated aerobic growth (Fig. 1A). Compared to aerobic growth, anaerobic growth of strain 10403s resulted in lower pH, higher concentrations of ethanol and lactic acid, and no detectable levels of acetoin (Table 1). Using TEM to visualize strain 10403s also highlighted a morphological difference between aerobically and anaerobically grown cells (Fig. 1B, C). Anaerobically grown strain 10403s exhibited a notably increased space between cytoplasm and the outer edge of the cells.

**Table 1:** Characterizations of *Listeria monocytogenes* strain 10403s in vitro growth

	Culture pH (BHI)	Culture pH (buffered BHI)	[Lactate] (mM)	[Acetoin] (mM)	[Ethanol] (%)
Aerobic	5.41 ± 0.14	6.57 ± 0.01	0	1.37 ± 0.51	0.22 ± 0.000
Anaerobic	4.67 ± 0.12	6.51 ± 0.03	1.75 ± 0.31	0	1.43 ± 0.002
*p* value^a^	0.002	0.48			0.009

Values shown are averages of triplicates ± standard deviation

^a^ *p* values were calculated between aerobic and anaerobic samples using a two-tailed student’s *t* test

### Effects of anaerobic exposure on cell culture infections

**Fig. 2:**
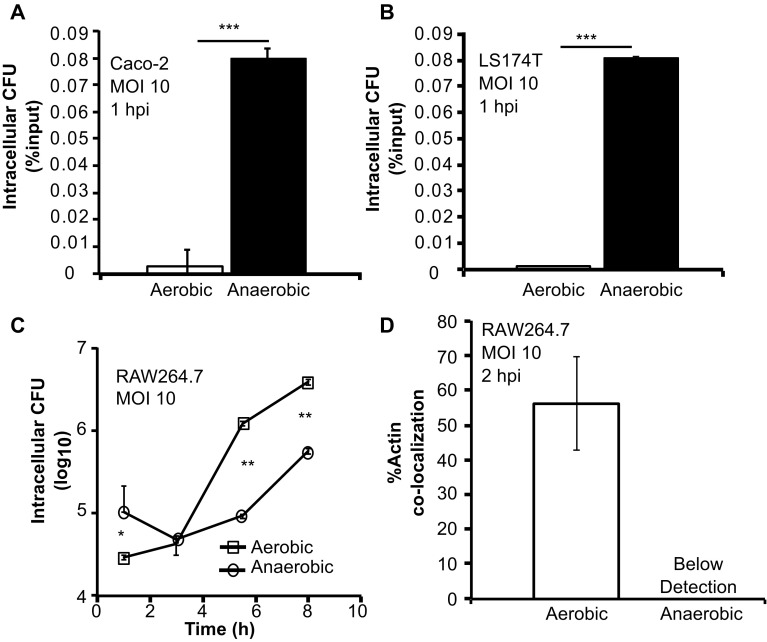
Anaerobic growth of *L. monocytogenes* leads to increased initial intracellular CFU but decreased intracellular growth and actin co-localization. Cell culture infections were performed with human colonic epithelial cell lines, Caco-2 (**A**) and LS174T (**B**), and with murine peritoneal macrophages, RAW264.7 (**C, D**). All infections were performed with MOI of 10 using aerobically or anaerobically grown *L. monocytogenes*. Approximately 100 *L. monocytogenes* cells were counted for actin co-localization per infection condition at 2 hpi. Averages of triplicates are plotted with *error bars* representing standard deviation and statistics were performed using a two-tailed student’s *t* test with significant differences indicated by asterisks (*p < 0.05, **p < 0.01, ***p < 0.001)

To determine the impact of anaerobic growth on *L. monocytogenes* infections, we infected murine macrophages (RAW264.7) and human colonic epithelial cells (Caco-2 and LS174T) with overnight *L. monocytogenes* grown under aerobic or anaerobic conditions. At 1 hpi, there was a significantly higher intracellular CFU in both Caco-2 (Fig. 2A) and LS174T (Fig. 2B) cells infected with anaerobically grown *L. monocytogenes* compared to those infected with aerobically grown bacteria. We also investigated the impact of anaerobic growth on infection stages beyond the initial invasion by monitoring intracellular growth of aerobically or anaerobically grown *L. monocytogenes* in RAW264.7 macrophages. While there was a higher number of intracellular bacteria in macrophages infected with anaerobically grown bacteria at 1 hpi, intracellular growth by anaerobically grown *L. monocytogenes* was significantly reduced in later timepoints post infection (Fig. 2C). Because intracellular growth relies on *L. monocytogenes* escape from phagosomes into the cytosol, we enumerated the proportion of cytosolic bacteria by measuring actin co-localization at 2 hpi inside macrophages. *L. monocytogenes* grown under anaerobic conditions exhibited significantly compromised actin co-localization compared to those grown under aerobic conditions (Fig. 2D). These data suggest that anaerobic growth has a strong effect on the outcome of infections. Moreover, because all infections were performed under aerobic conditions, the observed differences between aerobically and anaerobically grown bacteria suggest that anaerobic exposure prior to infections may have a long-term impact on subsequent interactions with host cells under aerobic conditions.

### Effects of anaerobic growth on LLO production

**Fig. 3:**
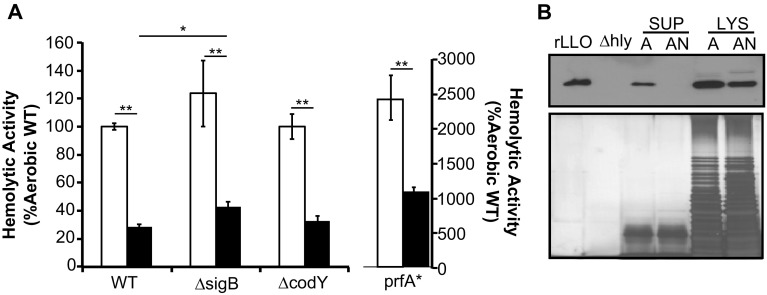
Anaerobically grown *L. monocytogenes* secretes less LLO. **A** LLO activity is decreased in anaerobic culture supernatant compared to aerobic culture supernatant of wildtype strain 10403s and isogenic mutants. Averages of triplicates are plotted with *error bars* representing standard deviation and statistics were performed using a two-tailed student’s *t* test with significant differences indicated by *asterisks* (*p < 0.05, **p < 0.01). **B** *top* Abundance of LLO is lower in anaerobic (“AN”) culture supernatant (“SUP”) compared to aerobic (“A”) culture supernatant. Lysate (“LYS”) of samples shows similar total protein levels. **B** *bottom* Silver stain was used as a loading control and shows similar total protein levels between aerobic and anaerobic samples. Recombinant LLO (“rLLO”) was used as a positive control and supernatant from mutant lacking the *hly* gene (∆*hly*) was used as a negative control

LLO is a secreted hemolysin and its pore-forming activity contributes to *L. monocytogenes* escape from phagosomes to the cytosol. Therefore, based on the infection phenotypes, we hypothesized that anaerobic growth, in contrast to enhancing invasion (Fig. 2A, B) (Bo Andersen et al. 2007; Burkholder et al. 2009), would cause a decrease in LLO production. We tested supernatant samples from overnight aerobic or anaerobic cultures for LLO activities through hemolytic assays and found little to no detectable hemolytic activity in the anaerobic culture supernatant (Fig. 3A). Using immunoblotting and silver staining, it was clear that while anaerobic growth did not alter the overall protein abundance in the supernatant (Fig. 3B, bottom), it resulted in a clear decrease in LLO abundance (Fig. 3B, top). Because LLO production can be regulated by multiple transcription factors—PrfA, SigB, and CodY (Rauch et al. 2005; de las Heras et al. 2011; Lobel et al. 2015), we tested isogenic mutants lacking known transcriptional regulators SigB (∆*sigB*) or CodY (∆*codY*) or harboring a constitutively active virulence master regulator PrfA (PrfA*) for their LLO production in response to anaerobic growth. While the PrfA* mutant exhibited higher levels of LLO production, all three mutants produced significantly lower levels of LLO under anaerobic conditions compared to aerobic conditions similarly to wildtype bacteria (Fig. 3D). These results highlighted that LLO production is under strong regulation by the presence or absence of oxygen. Moreover, this anaerobic suppression of LLO production is not directly mediated by known virulence regulators PrfA, SigB, and CodY.

### Effects of metabolic signals on anaerobic LLO production

**Fig. 4:**
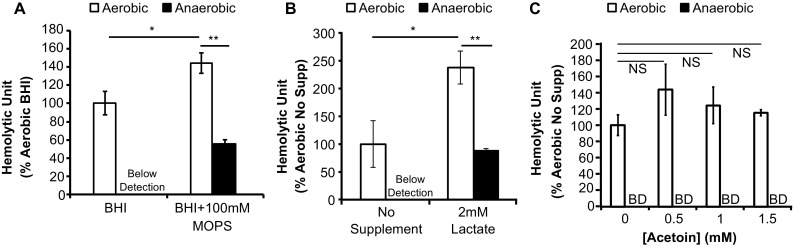
Buffering media or exogenous supplementation of lactate or acetoin does not alleviate the reduced LLO production under anaerobic conditions relative to aerobic conditions. **A** Compared to aerobic cultures, LLO activity in supernatant of anaerobic cultures in BHI or BHI buffered with MOPS (pH 7.0) was significantly lower. **B** Lactate supplementation enhanced culture supernatant LLO activity in aerobically and anaerobically grown *L. monocytogenes*. **C** Acetoin supplementation did not enhance LLO activity in aerobically or anaerobically grown *L. monocytogenes*. Averages of triplicates are plotted with *error bars* representing standard deviation and statistics were performed using a two-tailed student’s *t* test with significant differences indicated by asterisks (*p < 0.05, **p < 0.01). Samples with hemolytic activities less than half complete lysis are labeled as below detection (“BD”)

To identify factors contributing to regulation of LLO production in response to the presence or absence of oxygen, we investigated the role of physiological and metabolic signals differentially generated during aerobic or anaerobic growth. We first considered the role of lactic acid, a fermentation acid produced from pyruvate during *L. monocytogenes* anaerobic growth, in regulation of LLO production. The signal from lactic acid could be twofold—the acidification of the medium or the organic acid itself. To test the role of medium acidification, we measured LLO activity in the supernatant of cultures grown in buffered medium to prevent medium acidification with or without oxygen. In MOPS-buffered medium (pH 7.0), while there was no significant difference in pH between aerobic and anaerobic cultures (Table 1), LLO activity was significantly lower in anaerobic culture supernatant than that in aerobic culture supernatant (Fig. 4A). Exogenous supplementation of lactate (2 mM) resulted in increased LLO activity in both aerobic and anaerobic culture supernatants, but did not alleviate the relatively lower levels of anaerobic LLO production (Fig. 4B). In contrast, exogenous supplementation of the aerobic metabolite, acetoin, did not affect LLO activity in aerobic or anaerobic cultures (Fig. 4C). These results suggest that while acetoin and lactate are both metabolite products of pyruvate, only lactate supplementation influenced anaerobic LLO production.

### Effects of central carbon metabolites on LLO production

**Fig. 5:**
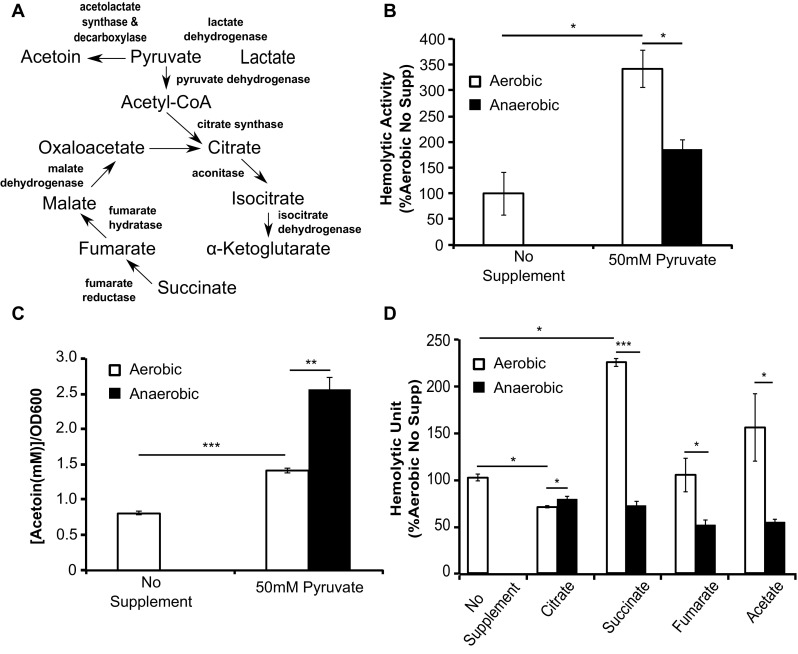
Supplementation of intermediates involved in central carbon metabolism alters carbon metabolism and increases supernatant LLO activity of anaerobically grown *L. monocytogenes*. **A** Simplified schematic shows three possible fates of pyruvate in *L. monocytogenes* central carbon metabolism. **B** Exogenous supplementation of pyruvate enhanced LLO activity in both aerobic and anaerobic culture supernatant. **C** Exogenous pyruvate supplementation increased acetoin concentrations in both aerobic and anaerobically grown *L. monocytogenes*. **D** Supplementation of intermediates of the TCA cycle (50 mM) enhanced anaerobic LLO activity. Averages of triplicates are plotted with *error bars* representing standard deviation and statistics were performed using a two-tailed student’s *t* test with significant differences indicated by asterisks (*p < 0.05, ***p < 0.001)

Lactate production is catalyzed by a reversible enzyme, lactate dehydrogenase, from pyruvate—a metabolite that connects to multiple carbon metabolic pathways in *L. monocytogenes* (Fig. 5A). Therefore, the effect of lactate on anaerobic LLO production is likely mediated by signals generated through pyruvate metabolism. When pyruvate was supplemented in the culture medium, we observed a dramatic increase in both aerobic and anaerobic LLO production (Fig. 5B). The pyruvate supplementation also resulted in an increase in acetoin production under both aerobic and anaerobic conditions (Fig. 5C), a phenotype suggesting exogenous pyruvate was taken up and metabolized. Because pyruvate is also metabolized to generate acetyl-coA for tricarboxylic acid (TCA) cycle, we tested the effects of TCA intermediates on anaerobic LLO production. If increase in the carbon flux through pyruvate was important in enhancing anaerobic LLO production, then supplementation of downstream metabolites in the TCA cycle should exhibit similar anaerobic enhancement of LLO production. Indeed, supplementations of acetate, citrate, succinate, and fumarate all resulted in higher levels of anaerobic LLO production (Fig. 5D). These data highlighted a potential role for central carbon metabolites in influencing LLO production in the absence of oxygen.

## Discussion

As an enteric pathogen, *L. monocytogenes* encounters fluctuating levels of oxygen from the aerobic oral cavity to the anaerobic intestinal lumen. As a result, metabolic adaptations to anaerobic conditions are an inevitable process during intestinal phase of infections. Here, we show that anaerobic growth resulted in major changes in carbon metabolism characterized by the lack of acetoin production and the increased production of lactate and ethanol. Ethanol concentrations for aerobic cultures may be underestimated because of the loss through culture agitation during aerobic growth. Curiously, anaerobic growth led to different morphologies under TEM. It is not clear if the differences in morphology are a result of specific structural differences or a result of different responses to TEM sample preparation processes. Nevertheless, both scenarios suggest surface modifications in anaerobically grown *L. monocytogenes* that can potentially lead to changes in stress resistance during transit through the anaerobic lumen and the intestinal phase of infections.

Anaerobic growth also resulted in significant changes in subsequent interactions with host cells under aerobic conditions. Anaerobically grown *L. monocytogenes* exhibited a significant increase in cell invasion but a significant decrease in actin co-localization and intracellular growth compared to aerobically grown bacteria. These results suggest that while anaerobic growth results in enhanced internalization into host cells, likely as a result of the increased expressions of internalins (Toledo-Arana et al. 2009) and LAP (Burkholder et al. 2009), it does not provide advantages in subsequent intracellular growth. Because *L. monocytogenes* entry into the host cytosol mainly relies on the activity of LLO (Hamon et al. 2012), the lack of actin co-localization phenotype can be partially attributed to the reduced LLO production exhibited by anaerobically grown bacteria. Alternatively, it is also possible that anaerobically grown *L. monocytogenes* have compromised intracellular expression of ActA, which facilitates actin polymerization as a means for bacterial motility and cell–cell spread. Given the role of *L. monocytogenes* dissemination in lethal infections, knowledge of how extracellular conditions influence subsequent intracellular behavior can be used to develop strategies to restrict *L. monocytogenes* infections in the intestines without spreading to peripheral organs.

To begin investigating the regulatory mechanism, we first tested the anaerobic LLO production in isogenic mutants either lacking known transcription regulators (∆*sigB* and ∆*codY*) or harboring constitutively active regulator (PrfA*). In all the mutants tested, hemolytic activities in anaerobic culture supernatant were significantly lower than those in aerobic culture supernatant. These results suggest that these known transcriptional regulators are not directly involved in the anaerobic suppression of LLO production. *L. monocytogenes* genome contains 15 putative members in the Crp/Fnr protein family (Glaser et al. 2001), which is known for their ability to detect and respond to environmental signals, such as fluctuating oxygen levels (Körner et al. 2003). Although mutations in each of these genes did not result in compromised growth in reduced oxygen conditions (Uhlich et al. 2006), these regulators may still play a direct or indirect role in detecting oxygen levels and modulating virulence gene expressions. In addition to the Crp/Fnr protein family, *L. monocytogenes* has 15 histidine kinases and 16 response regulators with demonstrated functions in fitness and pathogenesis (Flanary et al. 1999; Kallipolitis and Ingmer 2001; Cotter et al. 2002; Brøndsted et al. 2003; Kallipolitis et al. 2003; Dons et al. 2004; Williams et al. 2005; Larsen et al. 2006; Gottschalk et al. 2008; Collins et al. 2012; Nielsen et al. 2012; Vivant et al. 2014; Pöntinen et al. 2015). However, it is not clear how the signal transduction system is involved in *L. monocytogenes* anaerobic adaptations. Future investigations into their activities under anaerobic conditions can dramatically enrich our current understanding of *L. monocytogenes* anaerobic virulence regulation.

To further explore potential signals involved in the regulation of anaerobic LLO production, we first tested the effects of lactic acid, the main product of *L. monocytogenes* anaerobic metabolism, on anaerobic LLO production. We considered lactic acid as two separate signals, medium acidification and the organic acid itself, and found that the lower LLO production under anaerobic conditions compared to aerobic conditions cannot be explained by medium acidification or lactate. While lactate supplementation does not influence the potential suppression of anaerobic LLO production compared to aerobic LLO production, it enhances anaerobic LLO production compared to no lactate anaerobic control. This led us to consider anaerobic carbon metabolism as part of the signaling pathway leading to decreased anaerobic LLO production. Lactate is typically produced by *L. monocytogenes* from pyruvate through a reversible enzyme, lactate dehydrogenase. Therefore, the exogenous supplementation of lactate may potentially be converted back to pyruvate, which can then enter multiple carbon metabolic pathways. In contrast, the lack of effect from acetoin suggests that the acetoin production is a non-reversible pathway or that the expression of pathway enzymes is suppressed under anaerobic conditions. To directly confirm the role of pyruvate, we tested and demonstrated the positive effects of exogenous pyruvate on LLO and acetoin production. The dramatic effects of pyruvate observed in our study suggest that LLO production is sensitive to modulation by signals generated through pyruvate metabolism.

The TCA cycle is one of the main metabolic pathways utilizing pyruvate as the main carbon substrate. *L. monocytogenes* has an incomplete TCA cycle (Fig. 5A), lacking 2-oxoglutarate dehydrogenase, succinyl-CoA synthetase, and succinic dehydrogenase (Trivett and Meyer 1971; Glaser et al. 2001). Although an incomplete TCA cycle is not an uncommon genotype in bacteria (Huynen et al. 1999), its presence often demands additional means for bacteria to generate TCA intermediates to support anabolic pathways. *L. monocytogenes* is capable of generating oxaloacetate from pyruvate by pyruvate carboxylase (Schär et al. 2010) and succinate from γ-aminobutyrate (GABA) by the glutamate decarboxylase system coupled with the GABA shunt under acid stress conditions (Cotter et al. 2001; Feehily et al. 2013). As a result, the carbon flux of TCA cycle in *L. monocytogenes* might not be unidirectional and might change under different physiological conditions. In *E. coli* and *Bacillus subtilis*, TCA cycle is known to be suppressed under anaerobic conditions (Gray et al. 1966; Spencer and Guest 1987; Nakano et al. 1998) and by catabolite repression (Nakano et al. 1998; Gosset et al. 2004). While catabolite repression has been associated with *L. monocytogenes* virulence regulation (Gilbreth et al. 2004), which is known to respond to the presence of fermentable carbohydrates (Behari and Youngman 1998), the anaerobic TCA cycle activities have not been investigated in detail. If TCA cycle activity is reduced in *L. monocytogenes* under anaerobic conditions similarly to *E. coli* and *B. subtilis*, our results showing the positive effects of TCA cycle intermediates on anaerobic LLO production suggest a connection between the reduced TCA cycle activity and the decreased anaerobic LLO production.

All TCA cycle intermediates, when supplemented exogenously, resulted in an increase in anaerobic LLO production. Curiously, only citrate supplementation led to a significantly decreased aerobic LLO production compared to no supplementation control. Citrate has a multifaceted role in bacterial metabolism and physiology. As an intermediate metabolite in the TCA cycle, it serves as a feedback molecule that binds to the catabolite control protein C (CcpC) and suppresses the transcription of the first two genes in the TCA cycle—citrate synthase (*citZ*) and aconitase (*citB*) (Kim et al. 2006; Mittal et al. 2009). However, when the intracellular level of citrate is artificially high, as established with *citB* mutation, citrate-bound CcpC acts as a transcriptional activator for *citB* (Mittal et al. 2013). Therefore, the relationship between citrate levels and CcpC activities is not linear. It is possible that the opposing effects of exogenous citrate on aerobic or anaerobic LLO production reflect the different intracellular citrate levels achieved by exogenous citrate supplementations and the corresponding citrate synthase and aconitase activities under aerobic or anaerobic conditions.

In summary, our study highlights a critical role of anaerobic exposure in *L. monocytogenes* infections. *L. monocytogenes* grown anaerobically exhibit higher levels of internalization into host cells but compromised actin polymerization and intracellular growth, both of which might be attributed to the decreased LLO production. To better understand the mechanism underlying the anaerobic regulation of LLO production, our study suggests TCA cycle metabolites as positive signaling molecules for anaerobic LLO production. With anaerobic exposure a necessary step during infections, results from our study help strengthen current knowledge on *L. monocytogenes* adaptations and responses under anaerobic conditions.
